# Parasitological and molecular investigation of consequences of raw meat feeding (BARF) in dogs and cats: implications for other pets living nearby

**DOI:** 10.1007/s00436-024-08124-1

**Published:** 2024-01-29

**Authors:** Barbara Tuska-Szalay, Viktória Papdeák, Zsuzsanna Vizi, Nóra Takács, Sándor Hornok

**Affiliations:** 1 Department of Parasitology and Zoology, University of Veterinary Medicine, Budapest, Hungary; 2 Department and Clinic of Internal Medicine, University of Veterinary Medicine, Budapest, Hungary; 3 HUN-REN-UVMB Climate Change: New Blood-sucking Parasites and Vector-borne Pathogens Research Group, Budapest, Hungary

**Keywords:** RMBD, *Sarcocystis*, *Cystoisospora*, *Dicrocoelium*, *Eimeria*, Pseudoparasite

## Abstract

Considering the original nature of carnivorism among dogs and cats, nowadays these pets are increasingly fed BARF (Biologically Appropriate Raw Food/Bone And Raw Food) diet to improve their health conditions. However, this diet may also carry health risks, such as vitamin or mineral deficiencies, and infection with pathogens including parasites. In our study, fecal samples of 89 pet animals kept on BARF diet were subjected to coprological examination followed by molecular analyses. Six of them shed eggs of *Dicrocoelium dendriticum*. This result was confirmed with PCR and sequencing, and in one case, the DNA of *Fasciola hepatica* was also demonstrated. In addition, oocysts of *Cystoisospora canis*, a *Cystoisospora ohioensis*-like sp. and *Eimeria stiedai*, as well as sporocysts of a *Sarcocystis* sp. were also detected. All samples were negative for *Neospora caninum* and *Toxoplasma gondii*. In conclusion, no evidence was found for the infection of BARF-fed dogs and cats with parasites that are usually associated with this diet and considered as clinico-pathological risk factors for these pets themselves (e.g., *N. caninum*, *T. gondii*). However, fluke eggs (probably originating from ruminants) and oocysts of *E. stiedai* (from rabbit liver in the food) were demonstrated as pseudoparasites. These species are usually not considered among parasite-associated risks of BARF-feeding, implying that other animals living near BARF-fed pets are neglected in this context. However, where intermediate hosts of *D. dendriticum* occur in urban areas, BARF-feeding may indirectly affect later other dogs and cats. It was also shown here that BARF-feeding may contribute to the contamination of the environment with *E. stiedai* oocysts, thus increasing the risks of biliary coccidiosis in nearby living pet rabbits that would otherwise not have access to oocysts of *E. stiedai*.

**Graphical abstract:**

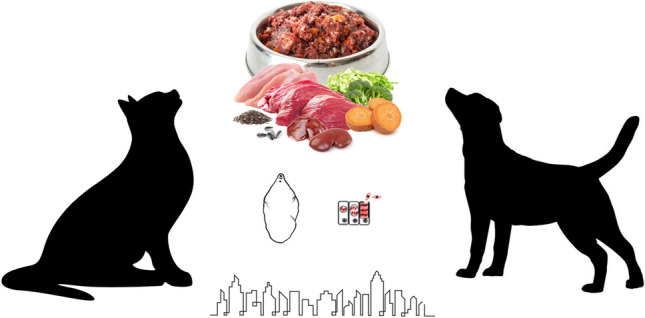

**Supplementary Information:**

The online version contains supplementary material available at 10.1007/s00436-024-08124-1.

## Introduction

Feeding dogs and cats with raw meat has recently shown an increasing trend among animal keepers. The raw meat-based diet (RMBD) or the BARF (Biologically Appropriate Raw Food/Bone And Raw Food) diet containing completely raw ingredients became more fashionable for various reasons, such as to revert to natural carnivorous lifestyle, to improve health conditions or simply because of discontent with commercial pet food.

Several studies reported the risks of BARF feeding, particularly deterioration in clinical conditions, i.e., hyperthyroidism, perforation of the gastrointestinal tract, and fracture of teeth (van Bree et al. 2018). Furthermore, consuming the raw meat several bacteria can occur in the feces of these animals, thereby increasing the risks to human and animal health. Concerning viruses Suide Herpesvirus 1 is worth mentioning which can cause the Aujeszky’s disease (pseudorabies) in dogs and cats. They are mostly infected by eating raw pork (Kölle and Schmidt 2015). The most common bacteria are *Salmonella* spp., *Campylobacter* spp., *Yersinia* spp., *Listeria monocytogenes*, and *Escherichia coli*. It is worth noting that the latter potentially antibiotic resistant *E. coli* might even trigger serious public health problems (Fredriksson-Ahomaa et al. 2017; van Bree et al. 2018).

On the other hand, BARF-fed pet animals can also become infected with various parasites. Considering apicomplexan protozoa, they might be potentially exposed to *Cryptosporidium* spp., *Cystoisospora* spp., *Toxoplasma gondii*, *Neospora caninum*, *Hammondia* spp., and *Sarcocystis* spp. (van Bree et al. 2018). Tissue cysts of *T. gondii* may occur in the most common meat-producing animals, such as poultry, cattle, sheep, and pig. While dogs are intermediate hosts of this parasite, cats can have copious oocyst shedding, thus they have epidemiological significance concerning both animals and humans. *Sarcocystis* tissue cysts may also occur in the striated muscle of omnivores and herbivores, and accordingly carnivores and, depending on *Sarcocystis* species, even human beings can acquire the infection by consuming cyst-containing meat. Dogs may also become final hosts in the life cycle of *N. caninum* and may pass oocysts after eating bovine placenta, fetus or minced meat of wild ruminants containing nerve tissues. In addition, dogs and cats may also harbor trematodes and cestodes, such as *Opisthorchis tenuicollis*, *Nanophyetus salmincola* and *Diphyllobothrium latum*, *Echinococcus granulosus*, respectively. Furthermore, nematodes may also affect these animals, i.e., *Anisakis simplex*, *Dioctophyma renale*, and *Trichinella* spp. (van Bree et al. 2018; Ahmed et al. 2021). *Echinococcus granulosus* has zoonotic potential and dogs might be infected by consuming viscera of the infected wild or domestic ungulates, e.g., pig and sheep (Jenkins et al. 2014). Last but not least, dogs and other carnivores are important reservoirs of zoonotic *Trichinella* spp. The main source of their infection might be the undercooked meat of domestic pigs, horses, or wild boars (Ahmed et al. 2021).

Taken together, the fact that raw meat might be contaminated with viruses, zoonotic bacteria, and parasites raises the question regarding the consequences of BARF diet (van Bree et al. 2018). In this study, our goal was to reveal the risks and the consequences of raw meat feeding in dogs and cats from a parasitological point of view. Therefore, we aimed to identify parasites found in the feces of dogs and cats fed with raw meat, to ascertain the source of their food, and to assess the epidemiological and public health significance of detected parasites.

## Materials and methods

### Sample collection

Eighty-one dogs and eight cats were involved in this study, all kept on BARF diet. From September 2021 to July 2022 fecal samples were collected from three different parts of Hungary: the region of Budapest, Central Transdanubia and Northern Great Plain.

### Parasitological analyses

Flotation (using 1300 g/l zinc-sulfate solution) was carried out on 3–5 g of the samples that arrived at the laboratory of Parasitology and Zoology Department of University of Veterinary Medicine Budapest. Diagnostic evaluation of samples and measurements were performed with a calibrated light microscope (Leica Microsystems, Wetzlar, Germany). Then, the fecal samples were stored at − 20 °C until further investigation.

### Molecular analyses

The DNA was extracted using the QIAamp Fast DNA Stool Mini Kit (QIAGEN, Hilden, Germany) according to the manufacturer’s instructions with slight modification. In particular, as the first step, mixtures of 300 mg fecal sample and InhibitEx solution were subjected to three freeze-thaw cycles, i.e., frozen overnight at − 80 °C and then kept at room temperature (20–25 °C) for 12 h. In our experience, this kit enables DNA extraction from coccidia even without freeze-thaw cycles if the number of oocysts/sporocysts is high (Tuska-Szalay et al. 2021), but the latter procedure was included to increase the breaking up of cyst walls of protozoan parasites and thus the efficacy of DNA extraction.

DNA extracts were stored at − 20 °C until they were molecularly analyzed by conventional PCRs for flukes, *Neospora caninum*, *Toxoplasma gondii*, *Cystoisospora* spp., piroplasms, and *Sarcocystis* spp. PCR components and cycling conditions are summarized in Supplementary Table 1, and the positive controls in Supplementary Table 2. The purification and sequencing of the PCR products were done by Biomi Ltd. (Gödöllő, Hungary). Obtained sequences were checked using the BioEdit program and then compared to GenBank sequences with the BLASTn program (https://blast.ncbi.nlm.nih.gov).

### Questionnaire-based data

Furthermore, questionnaires were filled out by the owners, to ascertain the source and type of raw meat or viscera given to the animals, whether the food contained nerve tissue, or if it was frozen before feeding. Additional questions referred to the usage of anthelmintics and signs of illness during the BARF diet.

## Results

### Parasitological analyses

**Fig. 1:**
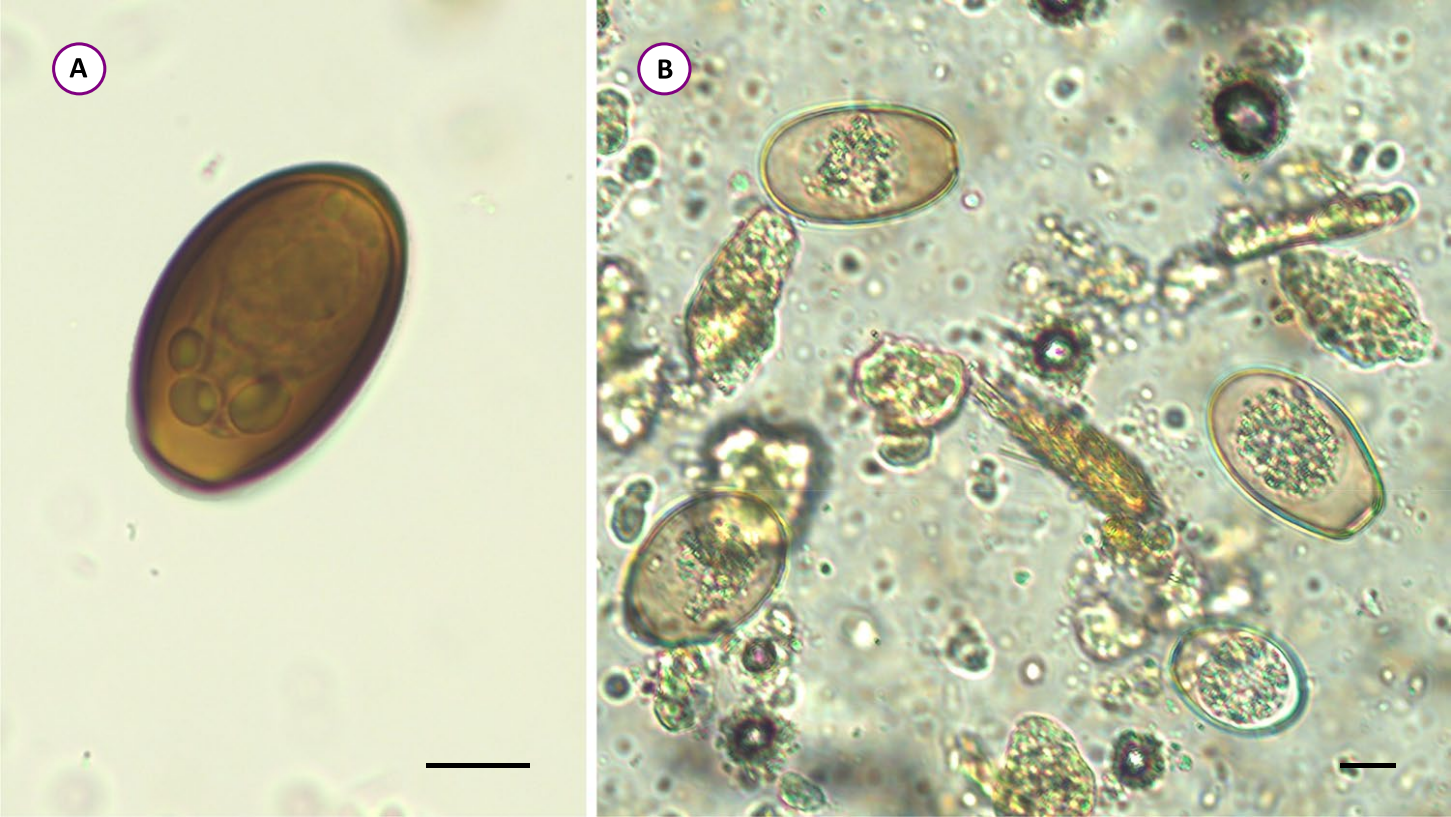
Pseudoparasites revealed from the feces of BARF-fed pets: **A** egg of *Dicrocoelium dendriticum*, **B** oocysts of *Eimeria stiedai* (larger) and *Cystoisospora ohioensis*-like sp. Bar = 10 μm

By microscopical examination, parasites were found in nine out of 89 animals. In the fecal samples of two dogs, oocysts of *Cystoisospora canis* (40 × 32 μm) (in BARF8), a *Cystoisospora ohioensis*-like sp. (25 × 16 μm), and *Eimeria stiedai* (35 × 20 μm) were detected (in BARF9) (Fig. 1B). In addition, in one sample (BARF76), sporocysts of a *Sarcocystis* sp. (13 × 7 μm) were seen. In six cases (three dogs: BARF2, BARF5, BARF41; and three cats: BARF1, BARF4, BARF64) *Dicrocoelium dendriticum* eggs (43 × 28 μm) were found (Fig. 1A).

### Molecular analyses

The samples examined for *Neospora caninum*, *Toxoplasma gondii*, *Cystoisospora* spp., *Babesia* spp., and *Sarcocystis* spp. were negative with molecular diagnostic methods. As a result of the PCR targeting the ITS2 gene of *Dicrocoelium* spp., six samples proved to be positive. The sequences from these samples (BARF1, BARF2, BARF4, BARF5, BARF41, and BARF64) showed 99.45–100% (376–378/378 bp) identity to a sequence available in GenBank from Italy (accession number: DQ379986.2). Furthermore, in the sample BARF41, beside *D. dendriticum*, the presence of *F. hepatica* was demonstrated by amplifying part of the cox1 gene. The corresponding sequence showed 100% (375/375 bp) identity to a conspecific sequence reported from cattle in Austria (MN507460.1). New sequences were submitted to GenBank (ITS2: OR539613-OR539615; cytochrome *c* oxidase subunit I, cox1: OR539617).

### Questionnaire-based data

Data of positive samples and some related information based on the questionnaire are summarized in Table 1. The most often fed contents were the meat/viscera of rabbit (66%), then cattle and lamb with 61%, fish and chicken with 57%. In addition, the owners fed their pet with duck, turkey, wild animals, and horse meat. Interestingly, seafood was given in the smallest percentage (1%). We received no usable information on whether any animals ate nerve tissue. Altogether, 96% of the owners froze the raw meat before feeding, and 49% of them used anthelmintic treatment every 3 months (against ascarid roundworms, hookworms, and tapeworms). The owners did not notice relevant symptoms during BARF diet.

**Table 1:** Detailing the results and the diet of the animals with positive samples

Sample	Parasitological examination (flotation)	PCR	Fed raw food	Frozen (− 20 °C) before feeding	Regular worming	Species
BARF1	+ (*D. dendriticum*)	+ (*D. dendriticum*)	Raw meat: beef, lamb, chicken, turkey, duck, fish Viscera: liver, spleen, kidney of ruminants	+	+	*Felis catus*
BARF2	+ (*D. dendriticum*)	+ (*D. dendriticum*)	Raw meat: beef, lamb, goat, rabbit, horse, game, goose Viscera: liver, spleen, kidney of ruminants and rabbit	+	+	*Canis lupus familiaris*
BARF4	+ (*D. dendriticum*)	+ (*D. dendriticum*)	Raw meat: beef, lamb, chicken, turkey, duck, fish Viscera: liver, spleen, kidney of ruminants	+	+	*Felis catus*
BARF5	+ (*D. dendriticum*)	+ (*D. dendriticum*)	Raw meat: rabbit, horse, goose Viscera: liver, spleen, kidney of rabbit, horse, goose Fruit, vegetable, dietary supplements	+	+	*Canis lupus familiaris*
BARF8	+ (*C. canis*)	-	Raw meat: lamb, chicken, rabbit, game, fish Viscera: liver, spleen, kidney of lamb, chicken	+	+	*Canis lupus familiaris*
BARF9	+ (*C. ohioensis*-like sp.*, E. stiedai*)	-	Raw meat: beef, lamb, rabbit, horse, duck, game Viscera: liver, spleen, kidney of ruminants and rabbit	+	+	*Canis lupus familiaris*
BARF41	+ (*D. dendriticum*)	+ (*D. dendriticum*, *F. hepatica*)	Raw meat: beef, chicken, turkey, fish Viscera: liver, spleen, kidney of cattle	+	+	*Canis lupus familiaris*
BARF64	+ (*D. dendriticum*)	+ (*D. dendriticum*)	Raw meat: beef, chicken, turkey, duck, rabbit, fish Viscera: liver, spleen, kidney, testis of ruminants and rabbit	+	+	*Felis catus*
BARF73	+ (*Sarcocystis* sp.)	-	Raw meat: beef, chicken Viscera: liver and brain stem of chicken	-	+	*Canis lupus familiaris*

## Discussion

In recent years, raw meat diet became more popular among pet owners, due to its potential beneficial effect on health. Consequently, research on the topic has increased and also reported the risks to parasitic infections related to raw meat feeding (Freeman et al. 2013).

Although dogs consuming raw meat are potentially exposed to the infection with *Neospora caninum*, the samples in our study were negative for this protozoon. It is worth to mention that shedding of *N. caninum* oocysts is often limited, therefore, false negative results can also occur. Serology is a more sensitive method. For instance, in a German survey, bitches were screened for *N. caninum* among which six seropositive dogs were fed with fresh raw meat (Villagra-Blanco et al. 2018). Furthermore, a recently published study analyzed commercial frozen RMBDs and reported the presence of *Sarcocystis cruzi*, *Sarcocystis tenella*, and *T. gondii* (van Bree et al. 2018). It is worth noting that the definitive host of *T. gondii* is the cat; however, interestingly, the DNA of this zoonotic protozoon has also been reported in dog feces (Zhu et al. 2022). This phenomenon might be related to coprophagia of feline feces, or to the ingestion of meat harboring *T. gondii* (Zhu et al. 2022), the latter relevant to BARF diet. The chance to detect its oocyst in cat feces is limited, since the oocysts are shed for relatively short periods of time (2–3 weeks) after infection, and the detection with microscope has low sensitivity. Although the presence of *Sarcocystis* spp. had been reported in Hungary (Szekeres et al. 2019; Tuska-Szalay et al. 2021; Hornok et al. 2015), we could not prove the presence of *Sarcocystis* spp*.* with molecular method, even though, we detected sporocysts of *Sarcocystis* in one sample. This might be explained by the low number of sporocysts. Furthermore, this was the only positive sample which was from a dog fed with fresh raw meat. Dogs can be infected with several *Sarcocystis* species, among which the most common species are *Sarcocystis cruzi* and *Sarcocystis tenella* (van Bree et al. 2018). In this study, the affected dog was fed with beef; hence, most probably it was infected by *S. cruzi*. Although sarcocystiosis is certainly associated with raw meat consumption considering the life cycle of this apicomplexan parasite, the clinic-pathological significance of this is low, because dogs as final hosts are usually symptomless (Saari et al. 2018).

Among coccidia, *Cystoisospora* spp. may infect dogs and cats and may cause gastrointestinal symptoms. As a result of a survey in 2001 in Hungary, where 490 dogs were screened for different parasites, the prevalence of *Cystoisospora* spp. was 3.5% (Fok et al. 2001). During the microscopical examination in the present study, two dogs (2.2%) were infected with *Cystoisospora* spp., i.e., one with *C. canis* and the other one is with a *C. ohioensis*-like species. Considering the latter, based on the morphological examination of the oocysts, it is difficult to distinguish *Cystoisospora burrowsi*, *Cystoisospora neorivolta*, and *C.ohioensis*, since they are similar and their size ranges overlap (Dubey 2019). The infection might have been associated with raw meat consumption, since monozoic tissue cyst of this protozoon can occur in paratenic hosts (Lindsay et al. 2014). However, we postulate that in the present cases the occurrence of *Cystoisospora* spp. was not associated with BARF-diet, because their prevalence was higher in the era preceding the widespread application of raw meat feeding (Fok et al. 2001).

In addition, oocysts of *E. stiedai* were also observed in one of the samples in coinfection with *C. ohioensis*-like species. *Eimeria stiedai* is the causative agent of biliary or liver coccidiosis of rabbits. It is an exceptional species in the strictly host-specific genus *Eimeria*, because it can infect both European brown hares (*Lepus europaeus*) and domestic rabbits (*Oryctolagus cuniculus*) (Varga 1976). Contamination of grass and other plants with *E. stiedai* oocysts may lead to transfer of the agent from wild to pet rabbits where these share habitats in rural areas (Bochyńska et al. 2022). However, pet rabbits in cities are kept in gardens or other types of enclosures where hares are excluded. According to the present results, however, even in highly urbanized areas there is a chance to contract *E. stiedai* by pet rabbits, e.g., when these are kept together (in the same household) or in the proximity of dogs fed by raw meat including rabbit liver. These dogs may pass *E. stiedai* from their food with their feces as pseudoparasites and may thus contaminate the environment of pet rabbits with sporulating *E. stiedai* oocysts. This may entail the consequences of biliary coccidiosis among pet rabbits, otherwise not having access to infectious oocysts of this *Eimeria* species. Furthermore, considering other pathogenic *Eimeria* species, rabbits can also be infected with *Eimeria* species causing intestinal coccidiosis, but as these species do not occur in viscera, they are unlikely to have relevance (as pseudoparasites) to BARF diet.

Last but not least, two fluke species (*F. hepatica*, *D. dendriticum*) that are mostly associated with ruminants as final hosts were also found in dog and cat feces. The eggs of *D. dentriticum* and presumably also of *F. hepatica* almost certainly originate from the BARF diet (in particular liver tissue of ruminants) and not from the infection of these pets, although they are also susceptible (Nesvadba 2006). This is also suggested by the comparison of the present results with those in the past when BARF feeding was not practiced in Hungary and no fluke eggs were recovered from the feces of dogs and cats in a large survey (Fok et al. 2001).

Further potential consequences of BARF feeding as shown in this study are relevant to dicrocoeliosis of dogs and cats. In contrast to the common liver fluke (*F. hepatica*) which has a life cycle bound to water, certain highly urbanized land-living snails are suitable first gastropod intermediate hosts of *D. dendriticum*, as exemplified by the common garden banded snail (*Cepaea hortensis*: Sánchez et al. 2021) and the Viennese banded snail (*C. vindobonensis*: Manga-González et al. 2001). The second intermediate hosts, *Formica* ants (Manga-González et al. 2001) also occur in urban, periurban areas (Seifert and Schultz 2009). Thus, although domestic ruminants, the principal final hosts of *D. dendriticum* are not kept (i.e., do not pass eggs) in urban areas, based on the relatively frequent *D. dendriticum* egg passage by BARF-fed pet animals in gardens and city parks we postulate that an urban cycle of the lanceolate fluke is likely to establish. If so, this may have serious implications on dogs and cats, because (1) they are well known for their grass-feeding habit (thus allowing ingestion of infected ants) and (2) clinical dicrocoeliosis is known to affect these pet animals severely: for instance, in cats, inappetence, diarrhea, and conjunctivitis, while in dogs loss of weight, vomiting, diarrhea, and skin lesions may occur (Nesvadba 2006).

Importantly, the above two risks apparently associated with raw meat feeding are usually not considered in its context (Ahmed et al. 2021). Neither *E. stiedai*, nor *F. hepatica* or *D. dendriticum* are listed among parasite-associated risks of BARF feeding, probably because usually self-inflicted harmful effects and infections are taken into account, both when components of the raw diet (van Bree et al. 2018) and when the pets fed with BARF are examined (Overgaauw 2020). Therefore, based on the present results, BARF feeding might promote the urban establishment of *D. dendriticum* that may affect later other dogs and cats (because intermediate hosts of *D. dendriticum* may occur in green areas of cities). At the same time, BARF-feeding may also increase the risks of biliary coccidiosis in pet rabbits living nearby which otherwise would not have access to oocysts of *E. stiedai*.

In summary, *E. stiedai*, *D. dendriticum*, and *F. hepatica* were detected as pseudoparasites in this study from dogs and cats kept on raw meat diet. Without analyzing the meat/viscera meant for consumption by dogs or cats, freezing for 2–3 days is strongly advised and this would kill any parasites potentially present (Ahmed et al. 2021). This method also has the advantage that the meat remains raw, fulfilling the criterion of BARF, but void of living parasites that would risk the health status of BARF-fed pets or others living nearby.

In conclusion, although the sample number of this study was limited, it revealed the potential significance of those parasites which are usually not considered from the point of view of the health status of raw meat-fed pets themselves. Since the eggs and oocysts that are found in the raw meat/viscera of various animals intended for consumption can have epidemiological importance after passing with the feces of BARF-fed dogs and cats, none of the pseudoparasites should be neglected in the context of BARF feeding.

### Supplementary information

ESM 1 (DOCX 31 kb)

ESM 2 (DOCX 16 kb)

## Data Availability

Sequences obtained in this study are deposited in GenBank under accession numbers OR539613-OR539615 (ITS2) and OR539617 (cox1). The authors confirm that all other data analyzed during the study are shown in the manuscript and its appendages or are available from the corresponding author upon request.
