# Supplementary material for: Parasitological and molecular investigation of consequences of raw meat feeding (BARF) in dogs and cats: implications for other pets living nearby
Source: Parasitol Res. 2024 Jan 29;123(2):114. doi: 10.1007/s00436-024-08124-1 (PMC10824860; doi:10.1007/s00436-024-08124-1)
Supplement: Supplementary file 1 — (DOCX 31 kb) [file 436_2024_8124_MOESM1_ESM.docx]

**Supplementary Table 1:** Primers and details for conventional PCR methods used in this study.

| **Target group** | **Target gene** | **Primer name** | **Primer sequence (5'-3')** | **Amplicon length (bp)** | **Thermocycling profile** | **Reference** |
| --- | --- | --- | --- | --- | --- | --- |
| **Flukes** | cox1 | JB3  JB4.5  mod-JB3  JB4.5 | TTT TTT GGG CAT CCT GAG GTT TAT  TAA AGA AAG AAC ATA ATG AAA ATG  TTT TTT GGG CAT AAT GAG GTT TAT  TAA AGA AAG AAC ATA ATG AAA ATG | ~450 | 95 °C for 5 min; 40× (95 °C for 40 s; 55 °C for 1 min; 72 °C for 1,5 min); 72 °C for 10 min | (Bowles et al. 1993)  (Wang et al. 2013) |
|  |  |  |  |  | 95 °C for 5 min; 40× (95 °C for 40 s; 51 °C for 1 min; 72 °C for 1,5 min); 72 °C for 10 min |  |
| ***Dicrocoelium* spp.** | ITS2 | 3S-fw  A28S-rev | GGT ACC GGT GGA TCA CTC GGC TCG TG  GGG ATC CTG GTT AGT TTC TTT TCC TCC GC | ~522 | 95 °C for 5 min; 40× (95 °C for 40 s; 55 °C for 1 min; 72 °C for 1,5 min); 72 °C for 10 min | (Prasad et al. 2007; Sahu et al. 2016) |
| ***Neospora* - *Toxoplasma* -**  ***Isospora* spp.** | COI | Toxo_COI_For  Toxo_COI_Rev | GGA GGA GGT GTA GGT TGG AC  CAT TTT GTA TTA TCT CTG GG | ~700 | 95 °C for 5 min; 40× (95 °C for 40 s; 55 °C for 30 s; 72 °C for 1,5 min); 72 °C for 10 min | (Ogedengbe et al. 2011) |
| **Neospora sp.** | NC5 | Np7  Np10 | GGG TGA ACC GAG GGA GTT G  TCG TCC GCT TGC TCC CTA TGA AT | ~200 | 95 °C for 5 min; 40× (95 °C for 40 s; 55 °C for 30 s; 72 °C for 1,5 min); 72 °C for 10 min | (Yamage et al. 1996; Wapenaar et al. 2006) |
| ***Toxoplasma* sp.** | repeat region | TOX-8 (fw)  TOX5 (rev) | CCC AGC TGC GTC TGT CGG GAT  CGC TGC AGA CAC AGT GCA TCT GGA TT | ~480 | 95 °C for 5 min; 35× (95 °C for 40 s; 60 °C for 1 min; 72 °C for 1 min); 72 °C for 10 min | (Homan et al. 2000; Reischl et al. 2003; Schares et al. 2008) |
| ***Piroplasma* spp.** | 18S rDNS | BJ1  BN2 | GTC TTG TAA TTG GAA TGA TGG  TAG TTT ATG GTT AGG ACT ACG | ~500 | 95 °C for 10 min; 40× (95 °C for 30 s; 54 °C for 30 s; 72 °C for 40 s); 72 °C for 5 min | (Casati et al. 2006) |
| ***Sarcocystis* sp.** | SSU | COC1*  COC2* | AAG TAT AAG CTT TTA TAC GGC T  CAC TGC CAC GGT AGT CCA ATA C | ~350 | 95 °C for 10 min; 40× (94 °C for 30 s; 54 °C for 30 s; 72 °C for 30 s); 72 °C for 10 min | (Ho et al. 1996) |

Bowles J, Hope M, Tiu WU, et al (1993) Nuclear and mitochondrial genetic markers highly conserved between Chinese and Philippine Schistosoma japonicum. Acta Tropica 55:217–229. https://doi.org/10.1016/0001-706X(93)90079-Q

Casati S, Sager H, Gern L, Piffaretti J-C (2006) Presence of potentially pathogenic Babesia sp. for human in Ixodes ricinus in Switzerland. Ann Agric Environ Med 13:65–70

Ho M, Barr B, Marsh A, et al (1996) Identification of bovine Neospora parasites by PCR amplification and specific small-subunit rRNA sequence probe hybridization. Journal of clinical microbiology 34:1203–8. https://doi.org/10.1128/JCM.34.5.1203-1208.1996

Homan WL, Vercammen M, De Braekeleer J, Verschueren H (2000) Identification of a 200- to 300-fold repetitive 529 bp DNA fragment in Toxoplasma gondii, and its use for diagnostic and quantitative PCR. Int J Parasitol 30:69–75. https://doi.org/10.1016/s0020-7519(99)00170-8

Ogedengbe JD, Hanner RH, Barta JR (2011) DNA barcoding identifies Eimeria species and contributes to the phylogenetics of coccidian parasites (Eimeriorina, Apicomplexa, Alveolata). Int J Parasitol 41:843–850. https://doi.org/10.1016/j.ijpara.2011.03.007

Prasad PK, Tandon V, Chatterjee A, Bandyopadhyay S (2007) PCR-based determination of internal transcribed spacer (ITS) regions of ribosomal DNA of giant intestinal fluke, Fasciolopsis buski (Lankester, 1857) Looss, 1899. Parasitol Res 101:1581–1587. https://doi.org/10.1007/s00436-007-0680-y

Reischl U, Bretagne S, Krüger D, et al (2003) Comparison of two DNA targets for the diagnosis of Toxoplasmosis by real-time PCR using fluorescence resonance energy transfer hybridization probes. BMC Infect Dis 3:7. https://doi.org/10.1186/1471-2334-3-7

Sahu R, Biswal DK, Roy B, Tandon V (2016) Molecular characterization of Opisthorchis noverca (Digenea: Opisthorchiidae) based on nuclear ribosomal ITS2 and mitochondrial COI genes. J Helminthol 90:607–614. https://doi.org/10.1017/S0022149X15000851

Schares G, Herrmann DC, Beckert A, et al (2008) Characterization of a repetitive DNA fragment in Hammondia hammondi and its utility for the specific differentiation of H. hammondi from Toxoplasma gondii by PCR. Mol Cell Probes 22:244–251. https://doi.org/10.1016/j.mcp.2008.04.003

Wang X-Y, Zhao G-H, Liu G-H, et al (2013) Characterization of Dicrocoelium chinensis from domestic yaks in Gansu and Sichuan provinces, China, using genetic markers in two mitochondrial genes. Mitochondrial DNA 24:263–266. https://doi.org/10.3109/19401736.2012.744974

Wapenaar W, Jenkins MC, O’Handley RM, Barkema HW (2006) Neospora caninum-like oocysts observed in feces of free-ranging red foxes (Vulpes vulpes) and coyotes (Canis latrans). J Parasitol 92:1270–1274. https://doi.org/10.1645/GE-913R.1

Yamage M, Flechtner O, Gottstein B (1996) Neospora caninum: specific oligonucleotide primers for the detection of brain “cyst” DNA of experimentally infected nude mice by the polymerase chain reaction (PCR). J Parasitol 82:272–279
