# Supplementary material for: Parasitological and molecular investigation of consequences of raw meat feeding (BARF) in dogs and cats: implications for other pets living nearby
Source: Parasitol Res. 2024 Jan 29;123(2):114. doi: 10.1007/s00436-024-08124-1 (PMC10824860; doi:10.1007/s00436-024-08124-1)
Supplement: Supplementary file 2 — (DOCX 16 kb) [file 436_2024_8124_MOESM2_ESM.docx]

| **Supplemantary Table 2.** Positive controls used in this study. | | | |  |  |  |
| --- | --- | --- | --- | --- | --- | --- |
|  |  |  |  |  |  |  |
|  | **Target group** | **Target gene** | **Primers** | **Positive controls** | **DNA code** | **Origin** |
|  | **Flukes** | cox1 | JB3 + JB4.5 | *Stichorchis subtriquetrus* | **BFL5** | beaver fluke |
|  |  |  |  | *Stichorchis subtriquetrus* | **BFL12** | beaver fluke |
|  |  |  | mod-JB3 + JB4.5 | *Stichorchis subtriquetrus* | **BFL5** | beaver fluke |
|  |  |  |  | *Stichorchis subtriquetrus* | **BFL12** | beaver fluke |
|  | ***Dicrocoelium* sp.** | ITS2 | 3S-fw + A28S-rev | *Stichorchis subtriquetrus* | **BFL5** | beaver fluke |
|  |  |  |  | *Dicrocoelium dendriticum* | **A1F** | dog feces |
|  | ***Neospora-Toxoplasma-Isospora* spp.** | COI | Toxo_COI_For + Toxo_COI_Rev | *Neospora caninum* | **Nc2** | tissue culture (kindly provided by Dr. Renate Edelhofer) |
|  |  |  |  | *Toxoplasma gondii* | **TACH Toxo** | tissue culture (kindly provided by Dr. Attila Sándor) |
|  | ***Neospora* sp.** | NC5 | Np7 + Np10 | *Neospora caninum* | **Nc1** | tissue culture (kindly provided by Dr. Renate Edelhofer) |
|  | ***Toxoplasma* sp.** | repeat region | TOX-8 (fw) + TOX5(rev) | *Toxoplasma gondii* | **TACH Toxo** | tissue culture (kindly provided by Dr. Attila Sándor) |
|  | ***Piroplasma* spp.** | 18S rDNA | BJ1 + BN2 | *Babesia canis vogeli* | **DOG/In7** | dog blood |
|  |  |  |  | *Babesia canis* | **MA14** | dog blood |
|  | ***Sarcocystis* sp.** | SSU | COC1* + COC2* | *Sarcocystis rileyi* | **SR5** | *S.rileyi* cyst from malards pectoral muscle |
